# Function of the Thyroid Gland

**Published:** 1887-08

**Authors:** 


### FUNCTION OF THE THYROID GLAND.

One of the most interesting series of researches regarding the function of the thyroid gland has been recently published by Prof. Herzen, of Lausanne.

From the results of numerous cases of removal of this body in connection with the physiological functions, as demonstrated by MM. Albertoni and Fizzoni, it is probable that the vexed question as to the use of this organ is now settled. The above named gentlemen believe that they have discovered the real function of this gland, and after a careful study of the blood of animals deprived of this gland, have come to the conclusion that it gives to hemaglobin the faculty of absorbing oxygen. The fact is, that the blood of animals which have been deprived of the thyroid gland contains a very small proportion of oxygen; their arterial blood contains less of this gas than the venous blood of healthy ones; and the investigators ascribe the symptoms of acute cachexia strumipriva in dogs to this very considerable diminution of oxygen which always followed upon enucleation of the gland.

The following case of removal of the thyroid gland, reported in the last number of the *London Lancet*, by James Gordon, M.D., is indicative of the correctness of Italian observations. Of the several cases reported, that of Mrs. A. will serve as a type. She was a widow, age forty-two, who had a large goitre of several years standing; operated upon by Prof. Lister eleven years ago; the entire thyroid gland completely excised; recovery from the operation perfect. The following characteristics were manifest in the course of the next few months: total change of voice, which could not be recognized by her own mother; her face was likewise so remarkably changed that no one knew her; it was of a sallow, pasty paleness, swollen about the eyelids, above and below, presenting a waxy, transparent look; the lips—formerly thin—were swollen and of a purple color; the entire expression had changed from that of natural brilliancy to almost imbecility; her manner of speaking had changed likewise—it was embarrassed and hesitating; the fingers became more gross and red, and were swollen; she could no longer button her dress nor perform any delicate operation with the hand—such as picking up small objects from the floor; the special sense of touch was gone; a general tendency to general paralysis ensued; the tongue became swollen; skin dry and rough; hair fell out; eyebrows became thin and scanty; memory impaired; the least pressure upon the skin left a livid mark behind, so that she was constantly ecchymosed about the hand.—*Indiana Medical Journal.*

